# Transcriptome Analysis of Storage Roots and Fibrous Roots of the Traditional Medicinal Herb *Callerya speciosa* (Champ.) ScHot

**DOI:** 10.1371/journal.pone.0160338

**Published:** 2016-08-03

**Authors:** Li Xu, Jiabin Wang, Ming Lei, Li Li, Yunliu Fu, Zhunian Wang, Mengfei Ao, Zhiying Li

**Affiliations:** 1 Institute of Tropical Crop Genetic Resources, Chinese Academy of Tropical Agricultural Sciences, Danzhou 571737, Hainan, China; 2 Ministry of Agriculture Key Laboratory of Crop Gene Resources and Germplasm Enhancement in Southern China, Institute of Tropical Crop Genetic Resources, Chinese Academy of Tropical Agricultural Sciences, Danzhou 571737, Hainan, China; Agriculture and Agri-Food Canada, CANADA

## Abstract

*Callerya speciosa* (Champ.) ScHot is a woody perennial plant in Fabaceae, the roots of which are used medicinally. The storage roots of *C*. *speciosa* are derived from fibrous roots, but not all fibrous roots can develop into storage roots. To detect key genes involved in storage roots formation, we performed Illumina sequencing of the *C*. *speciosa* storage roots and fibrous roots. *De novo* assembly resulted in 161,926 unigenes, which were subsequently annotated by BLAST, GO and KEGG analyses. After expression profiling, 4538 differentially expressed genes were identified. The KEGG pathway enrichment analysis revealed changes in the biosynthesis of cytokinin, phenylpropanoid, starch, sucrose, flavone and other secondary metabolites. Transcription factor-related differentially expressed genes (DEGs) were also identified, including such gene families as GRAS, COL, MIKC, ERF, LBD, and NAC. The DEGs related to light signaling, starch, sugar, photohormones and cell wall-loosening might be involved in the formation of storage roots. This study provides the first transcriptome profiling of *C*. *speciosa* roots, data that will facilitate future research of root development and metabolites with medicinal value as well as the breeding of *C*. *speciosa*.

## Introduction

*Callerya speciosa* (Champ.) ScHot is a woody perennial plant in *Fabaceae* and distributed over South China and Southeast Asia. Its roots have long been used for food and as a traditional medicinal herb, with properties of toxin removal, heat clearance from the lungs to relieve cough, liver purging and kidney invigoration. More recently, *C*. *speciosa* has been widely planted because the wild plants are on the brink of extinction, and rapid propagation has been applied for the manufacture-scale production of seedlings. In previous studies of *C*. *speciosa*, we have developed SSR markers to analyze population differences and diversity among natural populations, screened superior variety, and investigated the application of cryopreservation [1, 2]. The thickness and starch content are the main factors of quality of the storage roots. The storage roots (SRs) are string- or spindle-shaped and may be lignified and stop expanding under uncertain conditions. The FRs of *C*. *speciosa* possess the potential to form SRs, but limited FRs can transfer into SRs, the mechanism by which this occurs is poorly understood.

During evolution, some plants have acquired the ability to differentiate leaves, stems, or roots into storage organs in response to drought or freezing conditions [3]. The mechanisms of storage-organ formation have been investigated in root and tuber crops such as potato (*Solanum tuberosum*), sweet potato (*Ipomoea batatas*), cassava and yam. In particular, the tuberization mechanism has been most studied in potato. Homologs of the *flowering locus T* (*FT*) family are described as key components of signals inducing the formation of potato tuber and onion bulb [4, 5]. In potato, *CONSTANS* (*CO*) homologs repress the expression of mobile tuberizing signals under non-inductive long-day conditions (LD). *StCOL2*, which is expressed at higher levels in potato than other *CO* homologs, may repress tuberization by directly regulating the transcription of *StSP5G*, a putative tuberization repressor that may inhibits the expression of the mobile tuberizing signal *StSP6A* [4, 6]. In addition, potato homologs of *CYCLING DOF FACTOR 1* (*StCDF1*), *GIGANTEA* (*GI*) and *Flavin-binding kelch repeat F-box protein 1* (*FKF1*) form a complex that degrades *StCDF1*, thereby repressing the expression *StCOL1* and *StCOL2* and inducing tuber formation [7, 8]. Several plant hormones have been proven to be involved in tuberization in potato [9]. For instance, a decrease in the level of gibberellic acid (GA) is required for tuberization onset, indicating that GA plays an inhibitory role in tuberization [10, 11], and given the dramatic change in *StARF* and *StPIN* family gene expression during tuberization, it has been suggested that auxin functions as a promoter of tuber formation [12, 13]. By promoting cell division during tuberization onset and sink formation, cytokinins may also serve as universal regulators of storage-organ formation [14]. Some other mobile signals have been found to regulate tuberization in potato. miR156 and miR172, two major components of the flowering age pathway, were shown to travel to the stolon via the phloem and modulate tuber formation, with miR156 accumulating in stolons under LD and miR172 during tuberization onset [15, 16]. Overexpression of *StBEL5* and *POTATO HOMEOBOX1* (*POTH1*) in potato resulted in enhanced tuberization, and movement of the transcripts was demonstrated [17–19]. Furthermore, many tuberization-related transcription factors (TFs) such as NAC family genes, MADS-box family genes and knotted-like homeobox genes have also been found in sweet potato [20–25].

Several studies have applied transcriptome analysis to detect key genes involved in storage-organ formation of root and tuber crops. Firon *et al*. [26] performed transcriptome profiling of sweet potato roots, reporting up-regulated starch biosynthesis and down-regulated lignin biosynthesis in SRs compared to FRs. Sun *et al*. [27] studied tuberous root development in *Rehmannia glutinosa* using dynamic transcriptome profiling, and Shan *et al*. [28] used digital gene expression (DGE) tag profiling analysis to identify putative genes involved in photoperiodic tuberization in potato. Additionally, Sojikul *et al*. [29] revealed phytohormone action during cassava storage root initiation through genome-wide microarray analysis, and Yang *et al*. [30] employed expression profiling with microarray to demonstrate an active process of glycolysis/gluconeogenesis in cassava storage roots. As there is limited genomic and transcriptomic information for *C*. *speciosa* to date, in the present study, we applied Illumina RNA-seq to perform the first transcriptome profiling of *C*. *speciosa* roots to find candidate genes involved in SR formation and to provide sequence resources for further analysis.

## Results

### Sequencing and *de novo* assembly

To characterize transcriptome differences between SRs and FRs, total RNA was extracted in replicates to prepare cDNA libraries and then subjected to sequencing (S1 Fig). A total of 315,665,026 clean reads of 90 nt were generated (Table 1) and subsequently *de novo* assembled using Trinity, resulting in 161,926 unigenes with an N50 value of 2107 nt and a mean length of 1285 nt (Table 2). Using the CEGMA pipeline, we checked the completeness of the assembly by similarity searches of 248 conserved eukaryotic core genes [31]. The result indicated that 95.97% of the core genes were completely assembled and 3.81% were partially assembled, suggesting the completeness of the assembly.

**Table 1:** Statistics of reads.

	*Libraries*	*Number of Clean Reads*	*Total Length of Reads (nt)*
Storage Roots	SR1	54,454,978	4,900,948,020
	SR2	51,348,702	4,621,383,180
	SR3	51,218,046	4,609,624,140
Fibrous Roots	FR1	51,766,438	4,658,979,420
	FR2	54,840,008	4,935,600,720
	FR3	52,036,854	4,683,316,860

**Table 2:** Statistics of *de novo* assembly.

	*Libraries*	*Percentage of reads assigned to Unigenes*	*Number of Unigenes*	*Total Length*	*Mean Length*	*N50*
Storage Roots	SR1	58.74%	77,956	80,657,943	1039	1727
	SR2	56.74%	90,238	98,402,901	1090	1807
	SR3	54.34%	84,060	87,889,002	1046	1724
Fibrous Roots	FR1	57.32%	90,013	95,982,678	1066	1762
	FR2	53.95%	117,282,	119,457,513	1019	1750
	FR3	56.61%	92,268	99,420,748	1078	1774
	All	56.28%	161,926	208,090,130	1285	2107

### Functional annotation of the unigenes

**Fig 1:**
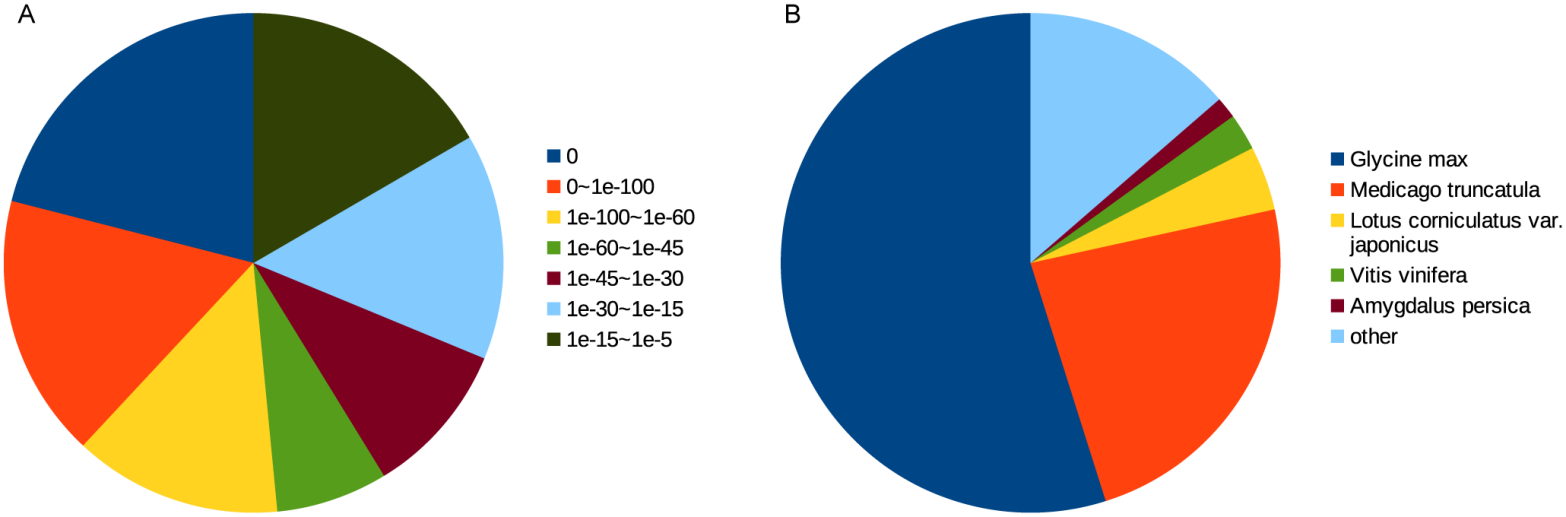
Summary of BLASTx results for the NR database. (A) E-value distribution of unigenes with BLAST hits in NR; (B) Species-based distribution of unigenes with BLAST hits in NR.

For functional annotations, the assembly unigenes were searched against the NCBI non-redundant protein (Nr), Gene Ontology (GO), Kyoto Encyclopedia of Genes and Genomes (KEGG), Cluster of Orthologous Groups (COG), NCBI Nucleotide (NT) and SwissProt databases using BLAST with an e-value cutoff of 1e-5. Of the 161,926 assembly unigenes, 125,967 matched to sequences in the databases. According to the BLAST results in the order of Nr, SwissProt, KEGG and COG, we predicted the coding sequences (CDSs) of 109,421 of the unigenes matching to sequences in the protein databases. Of those unigenes for which no sequence was matched, we predicted the CDSs of 8,159 using ESTScan. There are still 44,346 (27.39%) unigenes that have no CDS, some of which may function as non-coding RNAs. Based on the results of BLAST searches against the Nr database, 111,706 unigenes match to sequences of *Glycine max* (61,295), *Medicago truncatula* (26,330), *Lotus corniculatus* var. japonicus (4649), *Vitis vinifera* (2662), *Amygdalus persica* (1535), *Ricinus communis* (905) and other species (14,330) (Fig 1B). Of the 111,706 unigenes, 65,639 (58.76%) showed an e-value less than le-45, and 53,610 (47.99%) showed sequence similarity greater than 80% (Fig 1A).

**Fig 2:**
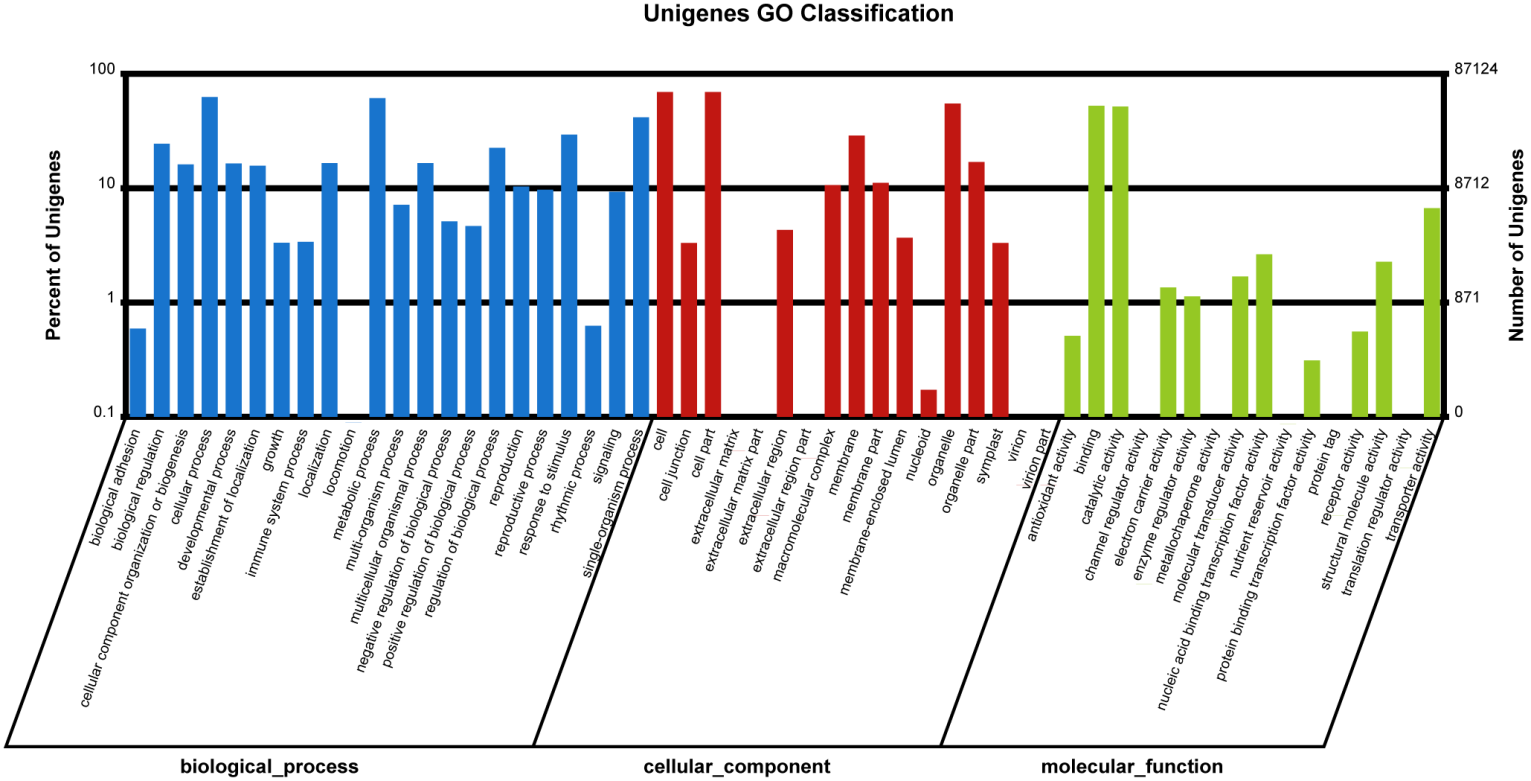
GO classification of unigenes. The number and percent of unigenes assigned to each GO category were provided in vertical axis.

To further characterize the function of unigenes, we applied GO functional classification to unigenes based on the results of BLAST against the Nr database. In total, 87,124 unigenes were classified into 56 functional groups according to the categories of biological process, cellular component and molecular function (Fig 2). Among the 22 functional groups in biological process, cellular process (54,563) and metabolic process (53,339) are the most highly represented groups, followed by single-organism process (36,337), response to stimulus (25,626), biological regulation (21,305), regulation of biological process (19,606), multicellular organismal process (14,433), localization (14,424), and developmental process (14,305), among others. Similar to biological process, unigenes were grouped into 16 sub-groups of molecular function, among which binding (45,752), catalytic activity (45,038), transporter activity (5818), nucleic acid binding transcription factor activity (2299), and structural molecule activity were the top 5 represented groups.

To further classify and predict functions, we also matched unigenes to the COG database (S2 Fig). A total of 47,162 unigenes were assigned to 25 functional categories: general function prediction only (15,610), transcription (9090), replication, recombination and repair (8366), signal transduction mechanisms (7114), posttranslational modification, protein turnover, and chaperones (5983), translation, ribosomal structure and biogenesis (5377), carbohydrate transport and metabolism (4884), and function unknown (4627), among others.

**Fig 3:**
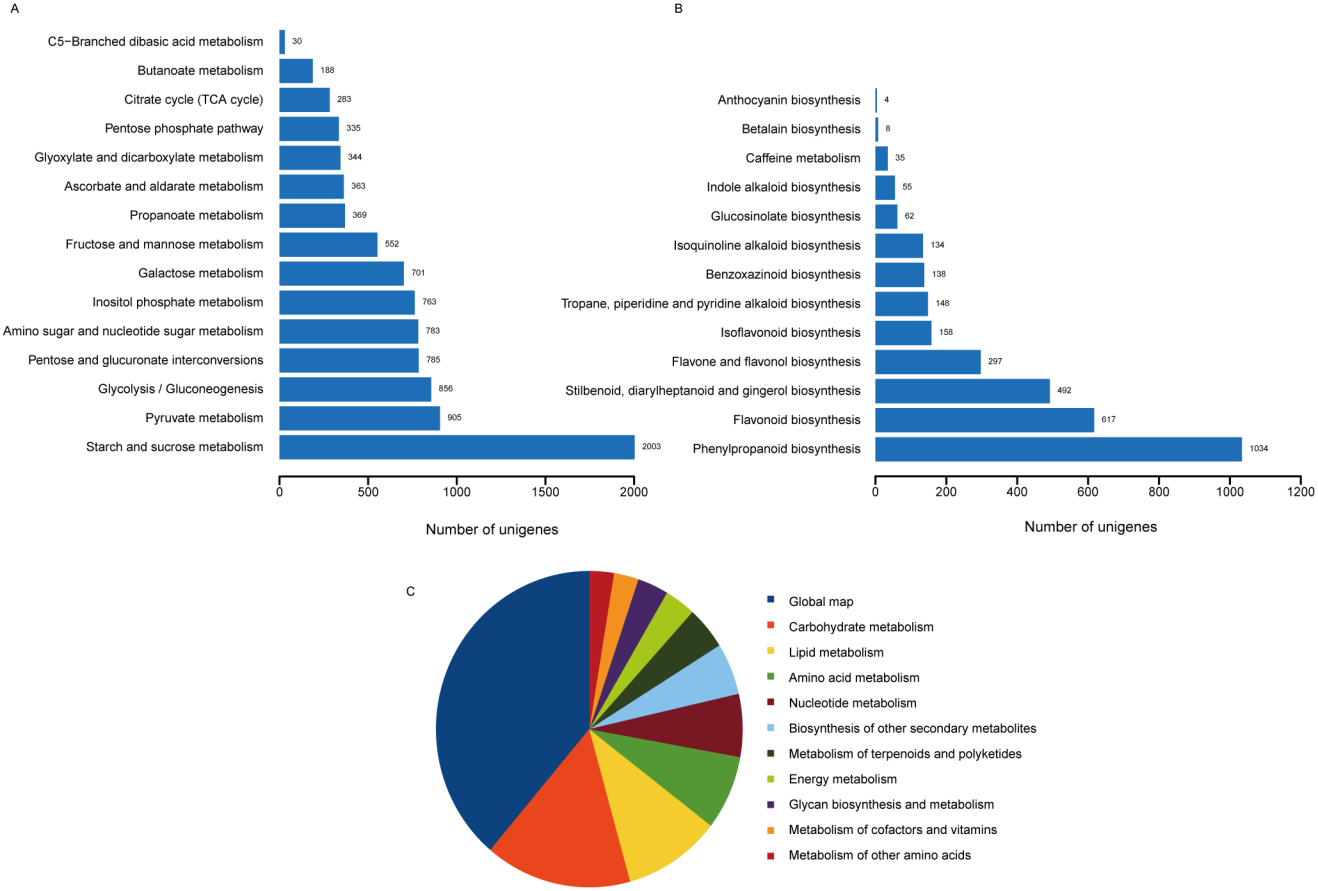
Summary of KEGG annotation of unigenes. (A) Number of unigenes involved in sub-pathways of carbohydrate metabolism. (B) Number of unigenes involved in sub-pathways of metabolism of other metabolites. (C) Number of unigenes involved in sub-pathways of metabolic pathway.

Based on a BLAST search against the KEGG database, 69,375 unigenes were assigned to KEGG pathways. Metabolic pathways and biosynthesis of secondary metabolites were the most represented pathways, followed by plant-pathogen interaction, plant hormone signal transduction, RNA transport, and spliceosome. Among metabolic sub-pathways, global map was the most represented, followed by carbohydrate metabolism, lipid metabolism, amino acid metabolism, nucleotide metabolism, and biosynthesis of other secondary metabolites (Fig 3C). For carbohydrate metabolism, sub-pathways of starch and sucrose metabolism, pyruvate metabolism and glycolysis/gluconeogenesis were the most highly represented (Fig 3A); phenylpropanoid biosynthesis, flavonoid biosynthesis, stilbenoid, diarylheptanoid and gingerol biosynthesis, flavone and flavonol biosynthesis and isoflavonoid biosynthesis were the top five represented sub-pathways of metabolism of other secondary metabolites (Fig 3B). Otherwise, some researches indicated that that many likely medical substances such as isoliquiritigenin, pterocapan, daucosterol, beta-Sitosterol, stigmasterol, chalcone, flavonoid, polysaccharide existed in roots of C. speciose, of which genes and pathways were identified in KEGG annotations [32, 33].

### Differential expression analysis

We first estimated the expression level of unigenes using fragments per kilobase of transcript per million fragments mapped (FPKM) values. The SR libraries and FR libraries contained 142,510, and 156,700 unigenes, with 19,416 specifically expressed in the SR libraries and 5,266 in the FR libraries. Using the NOIseq method proposed by Tarazona *et al*. [34] for differential expression analysis with |log_2_(SR/FR)|≥1 and probability≥0.8, we identified 4538 differentially expressed genes, among which 491 were up-regulated and 4047 down-regulated (S1 Table).

**Fig 4:**
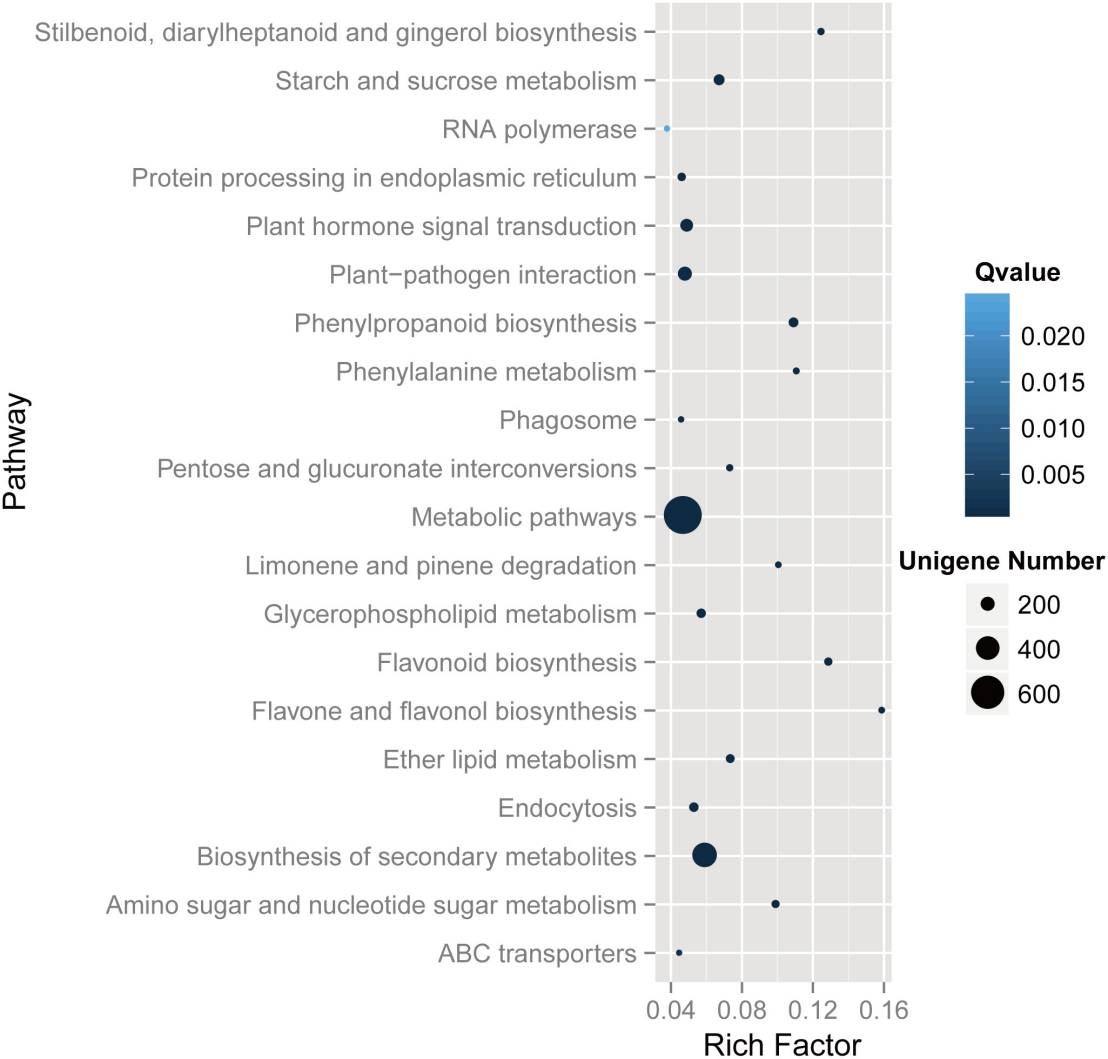
KEGG enrichment analysis of DEGs. The enriched kEGG pathways were with a q-value≤0.05. Enrichment factor: the ratio between the number of DEGs and all unigenes enriched in a particular pathway.

To explore the function of DEGs, 1579 of 4538 DEGs were assigned KEGG annotations and then subjected to KEGG pathway enrichment analysis. As a result, DEGs were classified to 118 pathways, 46 of which showed significant enrichment. Fig 4 illustrates the top 20 enrichment pathways. Metabolic pathways (699), Biosynthesis of secondary metabolites (420), and Plant-pathogen interaction (200) were the most common pathways, followed by Plant hormone signal transduction (172), Starch and sucrose metabolism (133), Phenylpropanoid biosynthesis (112), Endocytosis (104), Glycerophospholipid metabolism (102), Ether lipid metabolism (93), Protein processing in endoplasmic reticulum (84), and Flavonoid biosynthesis (79), among others.

### Identification of transcription factor genes among DEGs

**Fig 5:**
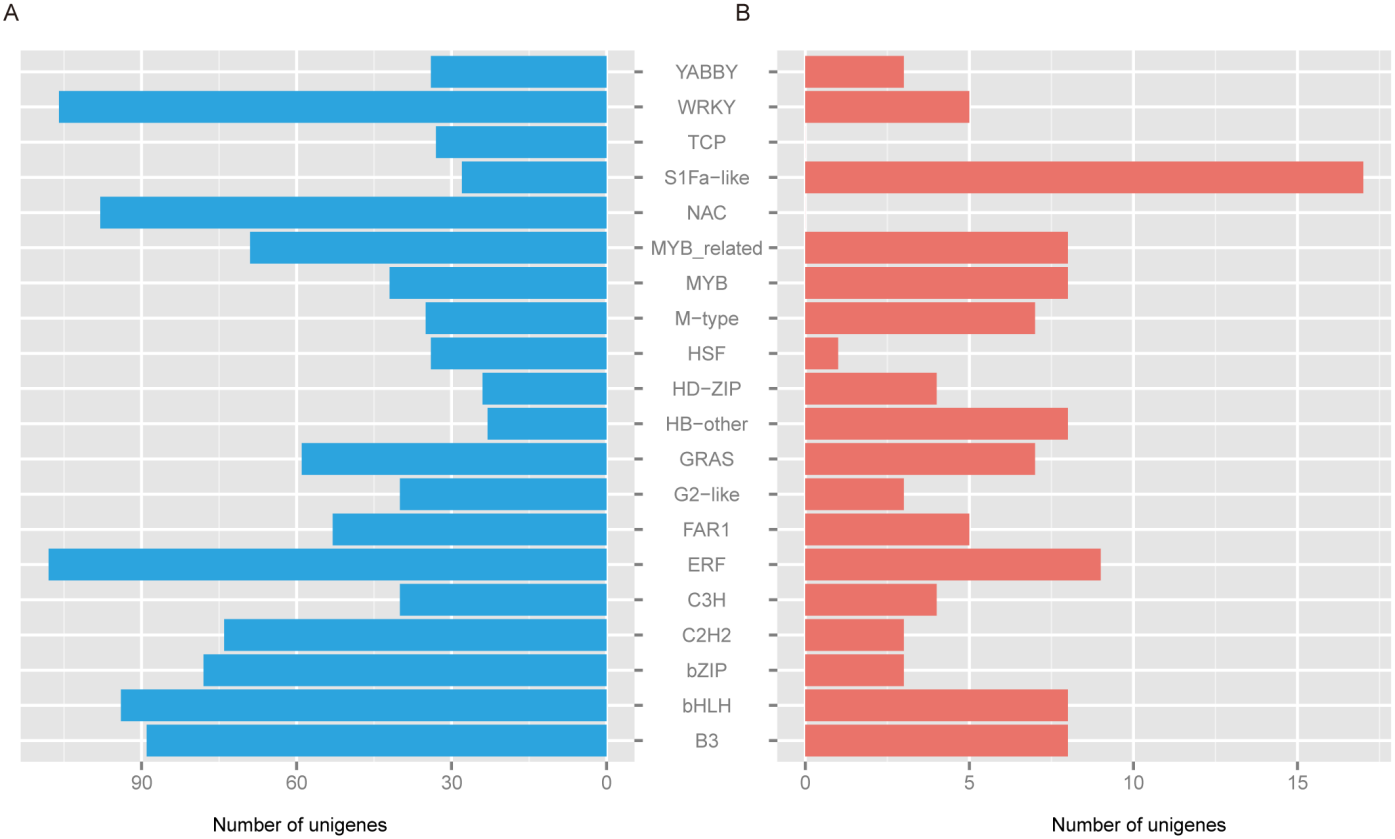
Summary of DEGs related to transcription factors. (A) Up-regulated unigenes related to transcription factors. (B) Down-regulated unigenes related to transcription factors.

To identify transcription factor (TF)-associated unigenes, BLASTx was used to search PlantTFDB for DEG sequences [35], with 1597 DEGs classified into 54 transcription factor gene families. The ERF (117) gene family was the most represented, followed by WRKY (111), B3 (110), bHLH (102), NAC (98), bZIP (81), MYB_related (78), C2H2 (77), GRAS (66), FAR1 (58), MYB (50), S1Fa-like (45), C3H (44), G2-like (43), and M-type (42), among others (Fig 5). Interestingly, the majority of the DEGs classified into transcription factor gene families were down-regulated DEGs, with 19 gene families having no up-regulated DEGs.

### Identification of DEGs involved in formation of storage roots

We identified DEGs might be involved in SR formation (Table 3, S2 Table). First, we examined unigenes might be involved in flowering timing, revealing homologs of *CO*, *FT*, *TERMINAL FLOWER 1* (*TFL1*), and *Flowering-promoting factor 1* (*FDF1*). Homologs of *FT* and *TFL1*, *CONSTANS-LIKE 2* and *CONSTANS-LIKE 5* were up-regulated, whereas homologs of *FDF1* and *EARLY FLOWERING 4* (*ELF3*) were down-regulated. Moreover, many TFs might be involved in flower and root development were identified. A large number of plant-specific transcription factors belong to NAC domain-containing protein families, some of which are involved in cell division and expansion, such as *NAC21/22*, *NAC29* and *NAC043*; these homologs were down-regulated in SRs. LOB-domain (LBD) genes comprise a large family of plant-specific and DNA-binding transcription factors, the functions of which are largely unknown [36], and homologs of *LBD4* and *LBD5* were up-regulated but homologs of *LBD13*, *LBD38* and *LBD41* down-regulated. *RAP1* belongs to the BHLH family of transcription factors, which are involved in anthocyanin biosynthesis and phytohormone signaling, and the homolog was found to be up-regulated in SRs. ARFs are transcription factors that response to auxin and function in promoting flowering, stamen development, and floral organ abscission as well as repressing cell division and organ growth [37]; homologs of *ARF4*, *ARF5*, *ARF6*, *ARF9* and *ARF18* were down-regulated. In the meanwhile, the homologs of auxin influx transporter *LAX1*, *LAX2*, *LAX3* and *LAX5* were down-regulated in SRs.

**Table 3:** Putative DEGs involved in storage root formation (Part of S2 Table).

*Unigene*	*Log2 FC*	*Probability*	*Homologue ID*	*E-value*	*Homolog name*
CL13177.Contig1_All	7.676971	0.969675	sp|Q00081|GLGL1_SOLTU	0	*Glucose-1-phosphate adenylyltransferase large subunit 1*
CL10402.Contig2_All	6.264433	0.953126	sp|P52417|GLGS2_VICFA	0	*Glucose-1-phosphate adenylyltransferase small subunit 2*
CL14063.Contig6_All	4.805002	0.939578	sp|P53536|PHSL_VICFA	0	*Alpha-1*,*4 glucan phosphorylase L isozyme*, *chloroplastic/amyloplastic*
CL12657.Contig1_All	3.97013	0.933003	sp|Q43092|SSG1_PEA	0	*Granule-bound starch synthase 1*, *chloroplastic/amyloplastic*
CL10444.Contig2_All	2.351379	0.886854	sp|Q0WVX5|SSY4_ARATH	3E-103	*Probable starch synthase 4*, *chloroplastic/amyloplastic*
CL11429.Contig2_All	-1.28089	0.815526	sp|Q8LCP6|GUN10_ARATH	0	*Endoglucanase 10*
CL13908.Contig14_All	4.335879	0.825065	sp|Q9LZS3|GLGB2_ARATH	0	*1*,*4-Alpha-glucan-branching enzyme 2–2*, *chloroplastic/amyloplastic*
CL886.Contig15_All	7.343399	0.928711	sp|P55231|GLGL3_ARATH	4E-89	*Glucose-1-phosphate adenylyltransferase large subunit 3*, *chloroplastic*
CL14396.Contig5_All	4.665488	0.934589	sp|Q94AZ2|STP13_ARATH	0	*Sugar transport protein 13*
CL1456.Contig8_All	1.190382	0.807632	sp|Q9SEK3|HXK1_SPIOL	0	*Hexokinase-1*
CL6463.Contig3_All	3.836333	0.928029	sp|Q9LPS1|HXK3_ARATH	0	*Hexokinase-3*
CL9768.Contig4_All	3.855858	0.917584	sp|Q38997|KIN10_ARATH	1E-133	*SNF1-related protein kinase catalytic subunit alpha KIN10*
CL9768.Contig3_All	2.961163	0.889714	sp|Q38997|KIN10_ARATH	2E-156	*SNF1-related protein kinase catalytic subunit alpha KIN10*
Unigene59772_All	-6.52946	0.831999	sp|P92958|KIN11_ARATH	5E-66	*SNF1-related protein kinase catalytic subunit alpha KIN11*
Unigene59262_All	-8.0805	0.940236	sp|P92958|KIN11_ARATH	9E-31	*SNF1-related protein kinase catalytic subunit alpha KIN11*
CL4742.Contig10_All	2.805577	0.854161	sp|Q9SYM4|TPS1_ARATH	0	*Alpha*,*alpha-trehalose-phosphate synthase [UDP-forming] 1*
CL4742.Contig7_All	1.85385	0.805582	sp|Q9SYM4|TPS1_ARATH	0	*Alpha*,*alpha-trehalose-phosphate synthase [UDP-forming] 1*
Unigene25815_All	-1.20994	0.813003	sp|Q9LMI0|TPS7_ARATH	9E-20	*Probable alpha*,*alpha-trehalose-phosphate synthase [UDP-forming] 7*
CL9903.Contig2_All	1.880861	0.86867	sp|Q9FUD3|BZIP9_ARATH	8E-52	*Basic leucine zipper 9*
Unigene33406_All	-3.26232	0.920375	sp|B9DGI8|BZP63_ARATH	1E-10	*Basic leucine zipper 63*
Unigene1547_All	-3.65527	0.924065	sp|B9DGI8|BZP63_ARATH	4E-12	*Basic leucine zipper 63*
CL6174.Contig7_All	1.380675	0.831663	sp|Q9LXL5|SUS4_ARATH	0	*Sucrose synthase 4*
CL6174.Contig5_All	-1.8308	0.861009	sp|O24301|SUS2_PEA	0	*Sucrose synthase 2*
CL11300.Contig1_All	1.980318	0.874639	sp|Q43876|SPSA_VICFA	0	*Probable sucrose-phosphate synthase*
CL11300.Contig2_All	1.668228	0.852002	sp|Q43876|SPSA_VICFA	0	*Probable sucrose-phosphate synthase*
CL12981.Contig1_All	-10.8547	0.993212	sp|O23547|EXLB1_ARATH	3.00E-89	*Expansin-like B1*
CL14227.Contig1_All	-4.78227	0.932096	sp|Q38865|EXPA6_ARATH	1.00E-130	*Expansin-A6*
Unigene25102_All	4.717632	0.938405	sp|O80622|EXP15_ARATH	2.00E-116	*Expansin-A15*
CL3091.Contig7_All	2.983223	0.909582	sp|O80622|EXP15_ARATH	1.00E-70	*Expansin-A15*
CL3091.Contig8_All	2.833029	0.90457	sp|Q9LDR9|EXP10_ARATH	7.00E-49	*Expansin-A10*
CL3091.Contig6_All	3.0807	0.916087	sp|Q9C554|EXPA1_ARATH	6.00E-71	*Expansin-A1*
Unigene41911_All	-2.86906	0.893997	sp|Q8LDW9|XTH9_ARATH	3.00E-57	*Xyloglucan endotransglucosylase/hydrolase protein 9*
Unigene40780_All	-3.84624	0.922292	sp|Q38857|XTH22_ARATH	4.00E-85	*Xyloglucan endotransglucosylase/hydrolase protein 22*
CL13489.Contig1_All	-4.17811	0.923414	sp|Q9ZSU4|XTH14_ARATH	9.00E-38	*Xyloglucan endotransglucosylase/hydrolase protein 14*
CL1947.Contig6_All	-1.50402	0.827194	sp|Q38909|XTH28_ARATH	6.00E-134	*Probable xyloglucan endotransglucosylase/hydrolase protein 28*
CL7466.Contig3_All	-2.06881	0.875615	sp|Q9SJL9|XTH32_ARATH	3.00E-123	*Probable xyloglucan endotransglucosylase/hydrolase protein 32*
Unigene37972_All	-1.24476	0.807754	sp|Q9C9Q8|PMTT_ARATH	5.00E-45	*Probable pectin methyltransferase QUA2*
Unigene21277_All	-1.42031	0.821245	sp|Q9LXK7|PME32_ARATH	2.00E-139	*Probable pectinesterase/pectinesterase inhibitor 32*
CL11485.Contig2_All	-1.70682	0.855194	sp|O81301|PME40_ARATH	8.00E-161	*Probable pectinesterase/pectinesterase inhibitor 40*
CL9924.Contig2_All	-1.77975	0.85495	sp|Q43111|PME3_PHAVU	0	*Pectinesterase 3*
Unigene17922_All	-1.8481	0.863072	sp|Q9FK05|PME61_ARATH	8.00E-71	*Probable pectinesterase/pectinesterase inhibitor 61*
Unigene36964_All	-1.91615	0.856509	sp|O04887|PME2_CITSI	0	*Pectinesterase 2*
CL9508.Contig4_All	-3.24505	0.921022	sp|Q1JPL7|PME18_ARATH	7.00E-172	*Pectinesterase/pectinesterase inhibitor 18*
CL11485.Contig3_All	-4.46623	0.926086	sp|O81301|PME40_ARATH	2.00E-106	*Probable pectinesterase/pectinesterase inhibitor 40*
Unigene66068_All	-3.75972	0.903985	sp|Q38890|GUN25_ARATH	8.00E-123	*Endoglucanase 25*
CL9596.Contig1_All	3.275602	0.922434	sp|Q9FS16|EXTN3_ARATH	1.00E-32	*Extensin-3*
CL9470.Contig2_All	-1.51796	0.821949	sp|Q9T0K5|LRX3_ARATH	0	*Leucine-rich repeat extensin-like protein 3*
CL13561.Contig2_All	-2.72166	0.896394	sp|O65375|LRX1_ARATH	4.00E-32	*Leucine-rich repeat extensin-like protein 1*
Unigene33730_All	-2.89631	0.913111	sp|Q9LUI1|LRX6_ARATH	7.00E-15	*Leucine-rich repeat extensin-like protein 6*
Unigene16895_All	-2.91198	0.898041	sp|Q9LJ64|PLRX1_ARATH	1.00E-111	*Pollen-specific leucine-rich repeat extensin-like protein 1*
CL13561.Contig1_All	-3.59081	0.926325	sp|Q9M1G9|EXTN2_ARATH	2.00E-103	*Extensin-2*
Unigene1149_All	-5.75528	0.948599	sp|Q9SN46|LRX5_ARATH	2.00E-31	*Leucine-rich repeat extensin-like protein 5*
Unigene26568_All	-1.662	0.845571	sp|O81081|LAC2_ARATH	9.00E-23	*Laccase-2*
Unigene47867_All	-9.01966	0.975263	sp|Q9FLB5|LAC12_ARATH	2.00E-64	*Laccase-12*
CL3996.Contig1_All	-3.93481	0.921552	sp|Q9SIY8|LAC5_ARATH	3.00E-145	*Laccase-5*
Unigene13230_All	-2.55428	0.898325	sp|Q9SR40|LAC7_ARATH	2.00E-104	*Laccase-7*
Unigene58553_All	-2.56409	0.895477	sp|Q9ZRF1|MTDH_FRAAN	1.00E-96	*Probable mannitol dehydrogenase*
Unigene37208_All	-2.15881	0.872666	sp|O82515|MTDH_MEDSA	0	*Probable mannitol dehydrogenase*

Many unigenes related to cell wall-loosening were found, such as homologs of expansins, extensins, pectin-related genes, xyloglucan endotransglucosylase/hydrolases and cellulase. However, only homologs of *Expansin-A15* (*EXPA15*), *Expansin-A10* (*EXPA10*), *Expansin-A1* (*EXPA1*) and *extensin-3* were up-regulated, whereas other genes related to cell wall-loosening proteins were down-regulated.

Furthermore, the expression levels of phytohormone biosynthetic genes were determined. Auxin, gibberellin, and abscisic acid biosynthesis-related genes, including homologs of *Abscisic-aldehyde oxidase*, *Flavin-containing monooxygenase YUCCA8*, *Indole-3-acetic acid-amido synthetase GH3*.*6*, *Jasmonic acid-amido synthetase JAR1*, *1-Aminocyclopropane-1-carboxylate synthase*, and *Gibberellin 20 oxidase 2*, were down-regulated. In contrast, a cytokinin biosynthesis-related unigene, a homolog of *Adenylate isopentenyltransferase 5*, was up-regulated.

Starch synthesis-related unigenes, such as homologs of *Starch-branching enzyme*, *Granule-bound starch synthase*, and *ADP-glucose synthase*, were also up-regulated. Additionally, sucrose-related unigenes homologous to *Sucrose synthase 4* (*SUS4*) and *Sucrose-phosphate synthase* (*SPS*) were up-regulated, whereas a homolog of *Sucrose synthase 2* (*SUS2*) was down-regulated. Moreover, unigenes linking sugar and development, including homologs of *Hexokinase-1* (*HXK1*), *SNF1-related protein kinase catalytic subunit alpha KIN10* (*SNRK1*.*1*), *Basic leucine zipper 9* (*BZIP9*), and *Alpha*,*alpha-trehalose-phosphate synthase [UDP-forming] 1* (*TPS1*), were up-regulated.

### qRT-PCR validation of DEGs

**Fig 6:**
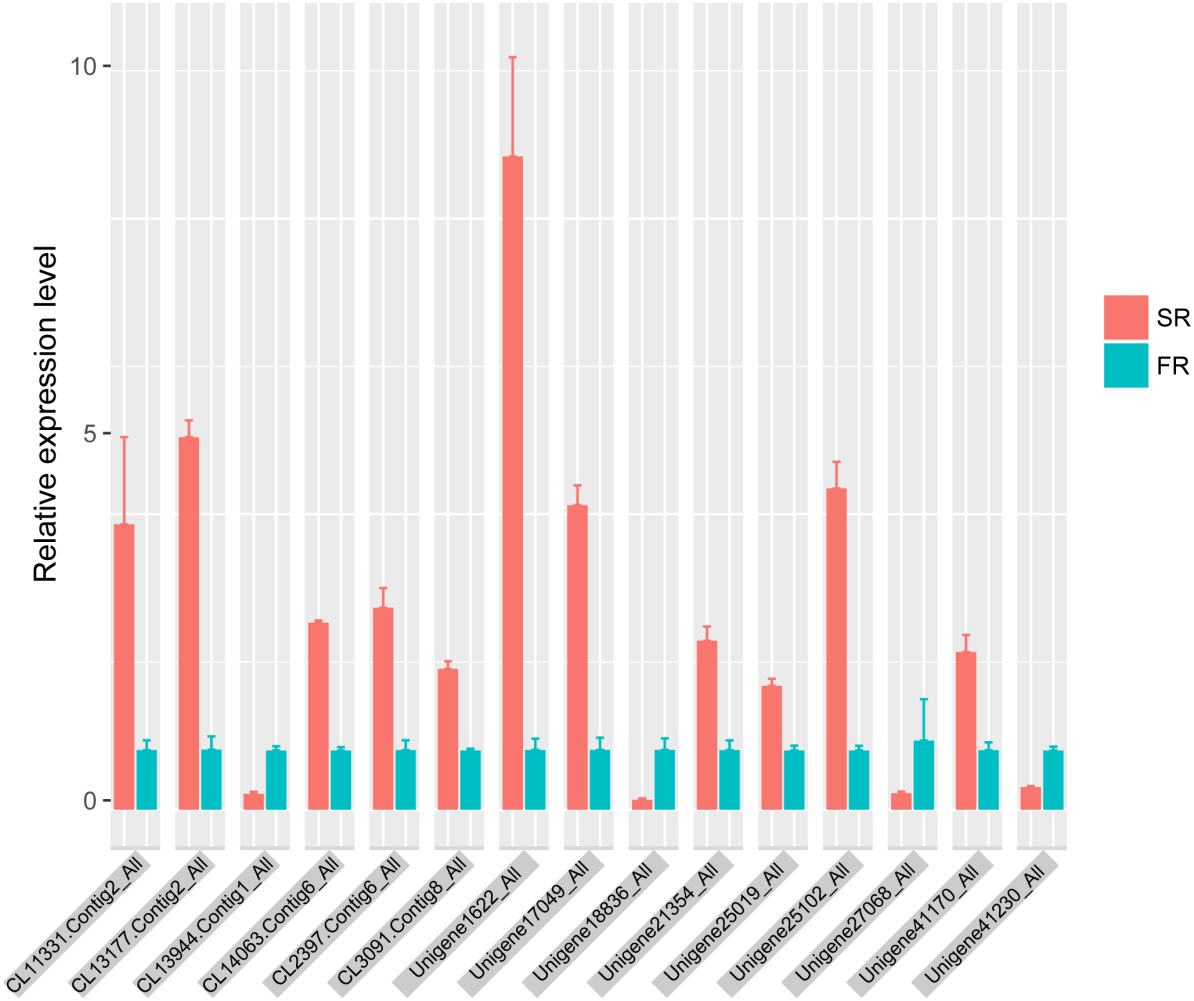
qRT-PCR validation of selected DEGs. SR, relative expression level of SR DEGs; FR, relative expression level of FR DEGs.

Fifteen DEGs were selected for qRT-PCR, all of which were found to be up-regulated or down-regulated in agreement with the differential expression analysis by RNA-seq (Fig 6). CL11331.Contig2_All, CL13177.Contig2_All, CL14063.Contig6_All and Unigene25102_All were up-regulated more than 25-fold in SRs, and the relative was up-regulated expression level less than 7-fold; the qRT-PCR-based results for Unigene41230_All, Unigene27068_All and Unigene18836_All also revealed a lower degree of relative down-regulation. This result suggests that high amounts of the unigene are not completely attributable to a high rate of gene expression.

## Discussion

This study provides a comparative transcriptome analysis between *C*. *speciosa* SRs and FRs. A total of 4538 DEGs were identified. KEGG pathway enrichment analysis revealed that DEGs were enriched in pathways such as starch and sucrose metabolism, plant hormone signal transduction, and phenylpropanoid biosynthesis, indicating changes in carbon flow and phytohormone signaling might happened during the formation of SRs of *C*. *speciosa*. DEGs were also enriched into the pathways Flavonoid biosynthesis, Flavone and flavonol biosynthesis, Isoflavonoid biosynthesis, Benzoxazinoid biosynthesis, Isoquinoline alkaloid biosynthesis, Indole alkaloid biosynthesis, and Glucosinolate biosynthesis, demonstrating the different secondary metabolite biosynthesis between SRs and FRs. Furthermore, DEGs related to transcription factors were predicted and involved many transcription factor gene families including bZIP, GRAS, COL, MIKC, ERF, LBD, NAC and HB-other families, which may participate in sugar and phytohormone signaling, flowering regulation, root development and cell expansion, indicating a wide range of transcription factors might be involved in the formation of SRs.

### Unigenes involved in light signaling and flowering

Appropriate photoperiod is acquired for initiation of storage organ in potato, lotus and onion [38–40]. And light is necessary for initiation of tuberous roots of *R*. *glutinosa*, with the intonation of tuberous roots were completely blocked when shaded with sunshade net [41]. Light signaling related genes *COL2*, *COL5*, *Early flowering 4 (ELF4)* and *serine/threonine protein kinase WNK8* differently expressed between SRs and FRs. *COL5* affects the expression FT and induce flowering in short-days [42], and *COL2* responds to light stimulus but not influence flowering in *A*. *thaliana* [43]. *ELF4* and *WNK8* control flowering time in *A*. *thaliana*, with *ELF4* involved in circadian rhythms and *WNK8* modulating photoperiod response [44, 45]. *FT* and *TFL1* both function in flowering time control and tuberization in potato, were up-regulated in SRs of *C*. *speciose* [4, 46]. Therefore, light signaling might be involved in the formation of SRs of *C*. *speciose*, and some flowering related genes expressed in roots may also have distinct functions in root development. Although the storage organs of *C*. *speciose*, *R*. *glutinosa*, potato, lotus and onion derive from different organs, similar regulation mechanisms may exist, on which light signaling might play a key role.

Large populations of mRNAs were found to be able to move between shoots and roots in Arabidapsis thaliana [47]. The mobility of mRNAs related to transcript abundance and half-life [48]. The mRNA of FT is involved in flowering induction by long distance movement in *A*. *thaliana* [49]. Some researches indicate that the expression of *FT* in underground stolons is activated by mobile *FT* protein in potato [4], which has not been found in other plants. However, there is no evidence that *FT* mRNA can transport from leaves into roots, although many mRNAs may able to move between shoots and roots in *C*. *speciose*. Therefore, further research is needed to determine whether the mRNA of *FT* in roots of *C*. *speciose* is transported from leaves or expresses locally.

### Unigenes related to sucrose and starch

A surplus of sugar in plants may be one of the main causes of storage root formation. Indeed, sugars can function as signaling molecular and gene regulators, regulating processes such as growth, flowering transition and tuber formation [50]. Sucrose has been found to promote tuber formation in potato, yam and *R*. *glutinosa* [51–53]. *Sucrose synthase* (*SUS*) converts sucrose into fructose and UDP-glucose, functioning as a sucrose-clearing enzyme [54]. Conversely, *Sucrose-phosphate synthase* (*SPS*), the key enzyme catalyzing the synthesis of sucrose, drives sucrose accumulation. The sucrose synthase alleles up-regulated in overall, for that homolog of *SUS4* showed an expression level 10-fold than homolog of *SUS2*, whereas *SUS4* up-regulated and *SUS2* down-regulated in SRs. The up-regulated sucrose synthase may also play a key role in the formation of SR of *C*. *speciose*.

The main form of carbohydrate stored by SRs of *C*. *speciose* is starch. The starch content of dried SRs is 49.3%, while dry matter content of the SRs is about 67% [55]. The storage organ of *C*. *speciose* and sweet potato both are modified roots that accumulated starch. The homologs of *ADP-glucose pyrophosphorylase* and *granule-bound starch synthase*, the key enzyme for starch synthesis, and *sucrose synthase* functioned in both sucrose synthesis and cleavage, up-regulated during SRs formation both in sweet potato and *C*. *speciose*, with similar expression pattern during SRs formation [26, 56]. The homolog of *beta-fructofuranosidase*, which functions as sucrose cleavage enzyme, showed a very low expression level, with a mean FPKM value of 0.16 and 0.07 in SRs and FRs of *C*. *speciose* respectively. The expression level of *beta-fructofuranosidase* of sweet potao were very low and decreased during SRs development [26, 56], whereas the expression level was higher in SRs than FRs in *C*. *speciose*, indicating that *beta-fructofuranosidase* may play different roles during SRs development between *C*. *speciose* and sweet potato. The transcriptome data also suggested that sucrose synthase was predominant sucrose cleavage enzyme for starch accumulation during SRs development of *C*. *speciose*, which is similar with sweet potato. Furtherly, study on proteomic of cassava during formation of tuberous roots indicated that *14-3-3 protein* up-regulated and play important roles in starch accumulation [57]. But homologs of *14-3-3 like protein C* and *14-3-3 protein 7* down-regulated in SRs, showing a possible and different role of *14-3-3 protein* during SRs formation and starch accumulation.

*Laccases*, which are involved in the lignin catabolic process, are required for secondary cell wall lignification [58, 59], and *Cinnamyl alcohol dehydrogenase* (*CAD*) also participates in lignin biosynthesis by catalyzing the final step of lignin monomer production. Homologs of *laccases* and *CAD* were down-regulated, indicating that lignin biosynthesis and lignification might decreased during SRs formation. The homolog of *Anthocyanin regulatory C1*, the key trans-acting factor required for anthocyanin biosynthesis, and the homolog of *Root-specific chalcone synthase* were both up-regulated in SRs, suggesting that changes in sugar metabolism may accompany changes in anthocyanin biosynthesis.

### Crosstalk between phytohormones, sugars and calcium signaling

Phytohormones play key roles in tuber or tuberous root formation. In potato, GA inhibits tuber formation and promotes stolon elongation [60]; in sweet potato, high cytokinin and auxin levels promote storage root initiation and growth [61, 62], and ABA levels positively correlate with the thickening of sweet potato storage roots [63]. GA-, ABA-, ethylene-, auxin-related biosynthesis unigenes were down-regulated, whereas cytokinin-related unigenes were up-regulated, indicating that cytokinin may participle in the formation of SRs in *C*. *speciosa*.

Plants possess systems that link carbon status and development, in wich the *Hexokinase* (*HXK*) play a key role. HXK-based signaling interacts negatively with auxin but positively with cytokinin [64, 65]. And *HXK1* signaling involves extensive crosstalk with plant hormone signaling via interaction with *F-actin* [66]. The up-regulation of homologs of *HXK1*, suggested that sugars may affect the formation of SRs partly via the regulation of complex networks of sugar-sensing systems in *C*. *speciosa*.

Sugar-based signaling pathways also interact with the calcium signaling pathway. *CIPKs* function together with calcium-sensing *CBLs*, comprising a *CPIK*-*CBL* network involved in plant signal transduction in response to abiotic stress [67]. *At*CIPK4 was found to be induced by salt stress, whereas *OsPK4*, the rice CIPK4 homolog, was induced by illumination, cytokinin treatment and nutrient deprivation [68, 69]. *AtCIPK7* is induced by cold and sugars and may function in sugar metabolism through the phosphorylation of sucrose synthase [67, 69]. In our study, homologs of *CIPK4* and *CIPK7* were up-regulated in SRs, indicating that *CIPKs* may also be involved in regulating the formation of SRs response environment and endogenous cues.

### Unigenes involved in cell-loosening and root development

Growth in cell volume begins with selective cell wall-loosening, in which cell wall stress is relaxed, resulting in consequential water uptake and cell enlargement [70]. The growing plant cell wall consists of one or more layers of cellulose microfibrils, with each microfibril directly contacting another or interacting with matrix polymers embedded in a hydrophilic matrix [71]. Expansins meditate acid-induced growth through non-lytic cell wall loosing. Based on sequence based phylogeny, plant expansins can be classed into two large families, *EXPA* and *EXPB*, and two small families, *EXPLA* and *EXPLB* [71]. In *C*. *speciose*, homologs of *EXPA1*, *EXPA10* and *EXPA15* are up-regulated and homologs of *EXPA6* and *EXPLB1* are down-regulated, of which the function need to character in the further research. Several transcription factors have been found to regulate expansins genes. *Arabidopsis thaliana homeobox 12* (*ATHB12*) increases the expression of *AtEXP10*, promoting cell expansion and leaf growth [72]. In SRs of *C*. *speciose*, *ATHB12* is down-regulated and the differential expression of expansins may attributable to the homologs of *ATHB12*.

It has been proposed that the root architecture influences the formation of tuberous root, with the development of lateral roots preventing lignification of the stele in the main axis of the adventitious root, which described in the swelling of sweet potato [26, 73]. LBD family genes are involved in root formation and are up-regulated in sweet potato tuberous roots [26, 74]. In addition, *LBD3* and *LBD4* are induced by cytokinin and required for a flat and asymmetrical leaf lamina in *Arabidopsis* [36]. *LBD4* may also be involved in SRs formation of *C*. *speciose*, with up-regulation of homologs of *LBD4* in SRs. *NAC21/NAC22* may function as a transcription activator mediating auxin signaling to promote lateral root development [75], and *NAC29* may function in the transition between active cell division and cell expansion [76]. *NAC043*, also called *NAC SECONDERY CELL WALL THICKENING PROMOTING FACTOR 1* (*NST1*), functions together with *NST2* and *NST3* in cell wall thickening and cell wall lignification [77–79]. The differences of root architecture between *C*. *speciose* SRs and FRs might partly resulted from the down-regulation of homologs of *NAC21*/ *NAC22*, *NAC29* and *NAC043* in SRs, which is need further research.

## Conclusion

We performed comparative analysis of *C*. *speciose* SRs and FRs, discovering a series of genes associates with light signaling, phytohormones, starch, sugar and cell wall-loosening differentially expressed between SRs and FRs, which may be involved in the formation of SRs. However, further research is required to determine the DEGs responsible for SR formation, which will shed lights on the mechanism of SR formation and help in the breeding of *C*. *speciose*.

## Methods

### Plant material

Three *in vitro*-grown seedlings transplanted in a greenhouse for 5 months were selected for the present study. The experimental site is the Institute of Tropical Crop Genetic Resources, Chinese Academy of Tropical Agricultural Sciences (CATAS). The greenhouse are on the conditions of temperature of 20–35°C, relative humidities of 85%-95%, transmittance of 20% and natural photoperiod. The three plants were planted in the nursery bed and watered and fertilized weekly. The potting soil was a mixture of equal parts coconut fibre, sand and turfy soil. The roots were sampled before fertilizing and watering at 9 am, when the soil was dry and the seedlings were exposed to light 2 h. The materials collected from each plant were grounded to fine powder using liquid nitrogen and mixed, and send to BGI Life Tech Co., Ltd (Shenzhen, China) for Illumina sequencing.

### RNA extraction, library preparation and sequencing

Total RNA was extracted using TRIzol^®^ Reagent (Invitrogen) following the manufacturer’s instructions. The integrity of the RNA samples was analyzed by agarose gel electrophoresis, and the purity and concentration were evaluated using an Agilent 2100 Bioanalyzer and NanoDrop (Thermo Scientific, USA). All RNA samples had an RNA integrity number (RIN) higher than 8. Libraries were prepared using Illumina TruSeq RNA Sample Preparation Kit (Illumina, USA) according to the manufacturer’s instructions. After checking quality and quantity, mRNA was isolated from total RNA using poly-(T) oligo-attached magnetic beads. The isolated mRNA was then sheared into short fragments by mixing with fragmentation buffer and used as templates for cDNA synthesis. cDNA was purified with a QIAquick PCR Purification Kit (QIAGEN), followed by end repair and acetylation of the 3’ ends. The Illumina pair-end adapters were then connected with the cDNAs. Finally, the cDNA libraries were checked using a Agilent 2100 Bioanalyzer and ABI StepOnePlus Real-time PCR system and sequenced using an Illumina HiSeqTM 2000 (Illumina Inc., San Diego, CA, USA). The sequence reads were submitted into the NCBI Short Read Archive (SRA) database and included in NCBI BioProject database with accession number PRJNA309919 (http://www.ncbi.nlm.nih.gov/bioproject/309919).

### *De novo* assembly and gene annotation

After the filtering of low-quality reads and removing adaptors, clean reads were assembled using Trinity *de novo* transcriptome assembly [80]. The unigenes in each sample were processed for sequence splicing and redundancy removal to generate a non-redundant unigene dataset. The core eukaryotic gene-mapping approach (CEGMA) was used to assess the completeness of the *de novo* assembly. The unigenes were then searched against protein and nucleotide databases, including SwissProt, NCBI's non-redundant protein database (NR), NCBI's non-redundant nucleotide database (NT), Kyoto Encyclopedia of Genes and Genomes (KEGG) and COG (Cluster of Orthologous Groups) using BLASTX with an e-value cutoff of 1e-5. The unigenes were annotated by GO with Blast2GO sing the BLAST results for NR [81]. The unigenes were assigned with KEGG annotations using the BLAST results for KEGG [82]. Based on the results of protein database searches, the sequence direction and coding region were determined for unigenes with BLAST hits; ESTscan was used to predict coding regions for those without BLAST hits [83].

### Unigene expression analysis

FPKM was calculated to assess unigene expression. NOISeq, a nonparametric approach, was used to determine the DEGs of samples with replicates, with a fold change≥2 and probability≥0.8 [34]. To characterize the function of DEGs, KEGG pathway enrichment analysis was performed using KOBAS 2.0 (q-value≤0.05) [83]. DEGs were also searched in PlantTFDB to determine DEGs related to TFs [35].

### Validation of qRT-PCR

Fifteen DEGs were selected for qRT-PCR validation of gene expression. Gene-specific primers were designed with PRIMER 6.0 software (University of Plymouth); the primers are listed in S3 Table. Total RNA was extracted from SRs and FRs described as above. The total RNA were treated with DNaseI (Takara).With validation of the expression level of several reference genes using qRT-PCR and expression stability analysis the reference genes by Genorm (https://genorm.cmgg.be) [84], the *Actin-2* gene was used as an internal control (S3 Fig). The standard curve of each selected DEG was obtained by qRT-PCR with a series of cDNA dilutions. The 10 μl reaction mixture for qRT-PCR consisted of 5 μl of 2× SYBR Green Master Mix Reagent (Applied Biosystems), 0.2 μM of gene-specific primers and 50 ng of cDNA sample. The amplification reactions were performed with following program: 95°C for 10 min and 45 cycles of 95°C for 5 s and 60°C for 30 s. The relative expression levels of DEGs were calculated with the 2^−△△Ct^ method. qRT-PCR was performed with 3 biological replicates and 3 technical replicates for each experiment.

## Supporting Information

S1 Fig Seedlings for RNA-seq. (JPG)

S2 Fig COG functional classification of unigenes. (TIF)

S3 Fig Average expression stability of refrence genes. ACT2: Actin-2; ACT7: Actin-7; REFA1: Elongation factor 1-alpha; TUBA1: Tubulin alpha-1 chain; UBQ10: Polyubiquitin 10.
(TIF)

S1 Table DEGs between storage roots and fribrous roots. (XLS)

S2 Table Putative DEGs involved in storage root formation. (DOC)

S3 Table Summary of qRT-PCR validation. (DOC)
