# Supplementary figures and images for: Transcriptome Analysis of Storage Roots and Fibrous Roots of the Traditional Medicinal Herb Callerya speciosa (Champ.) ScHot
Source: PLoS One. 2016 Aug 3;11(8):e0160338. doi: 10.1371/journal.pone.0160338 (PMC4972434; doi:10.1371/journal.pone.0160338)

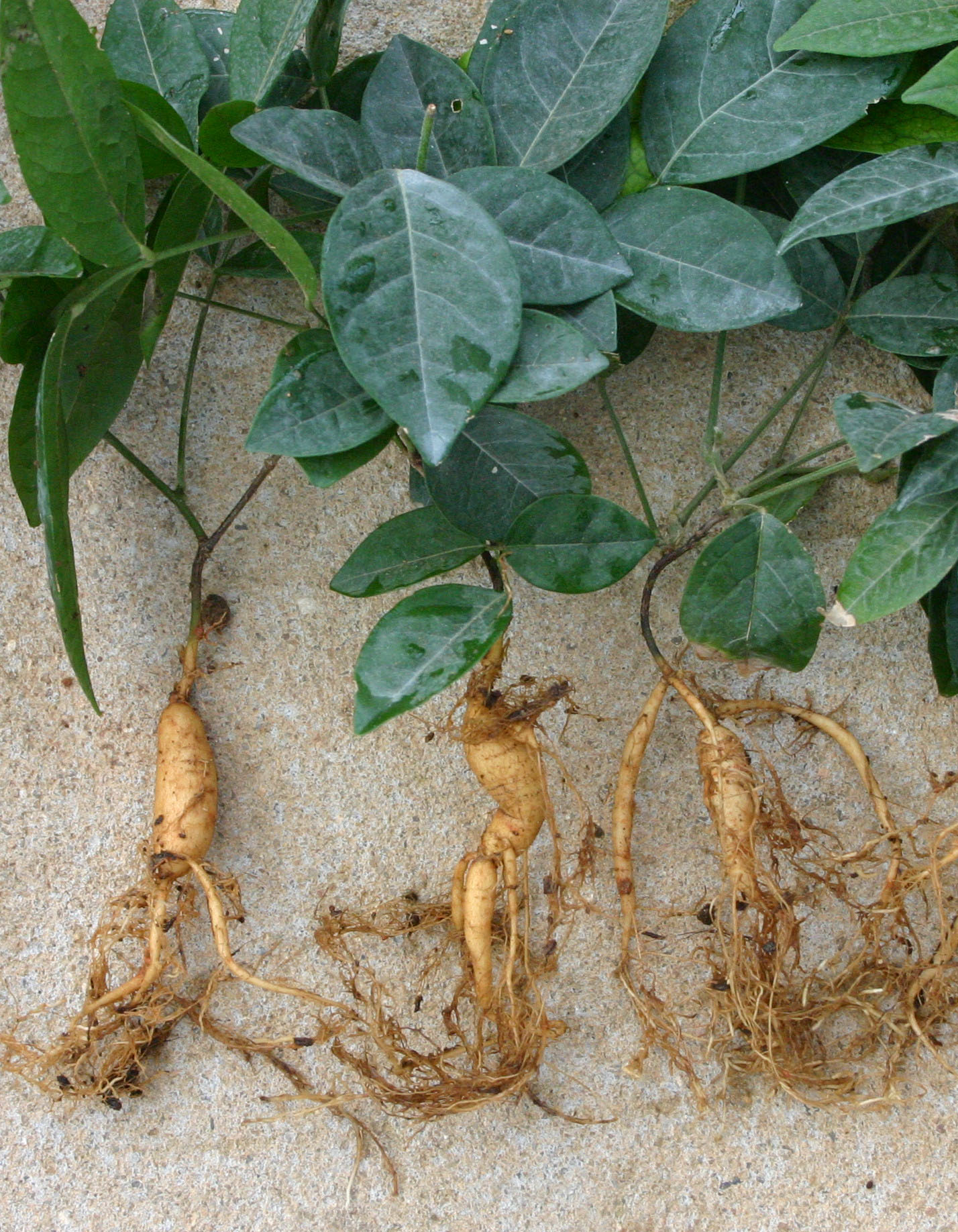

Supplement: S1 Fig — (JPG) [file pone.0160338.s001.JPG]

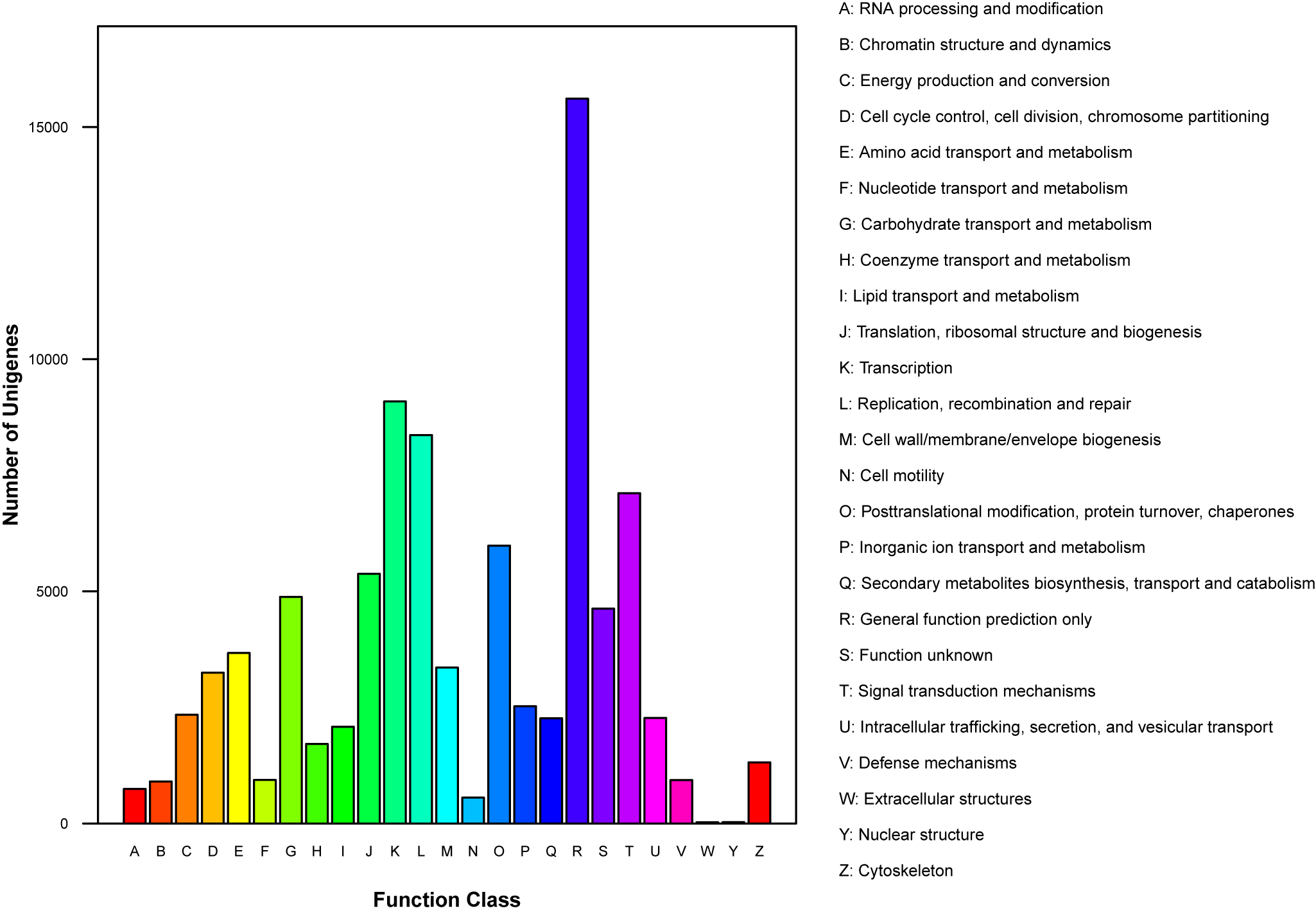

Supplement: S2 Fig — (TIF) [file pone.0160338.s002.tif]

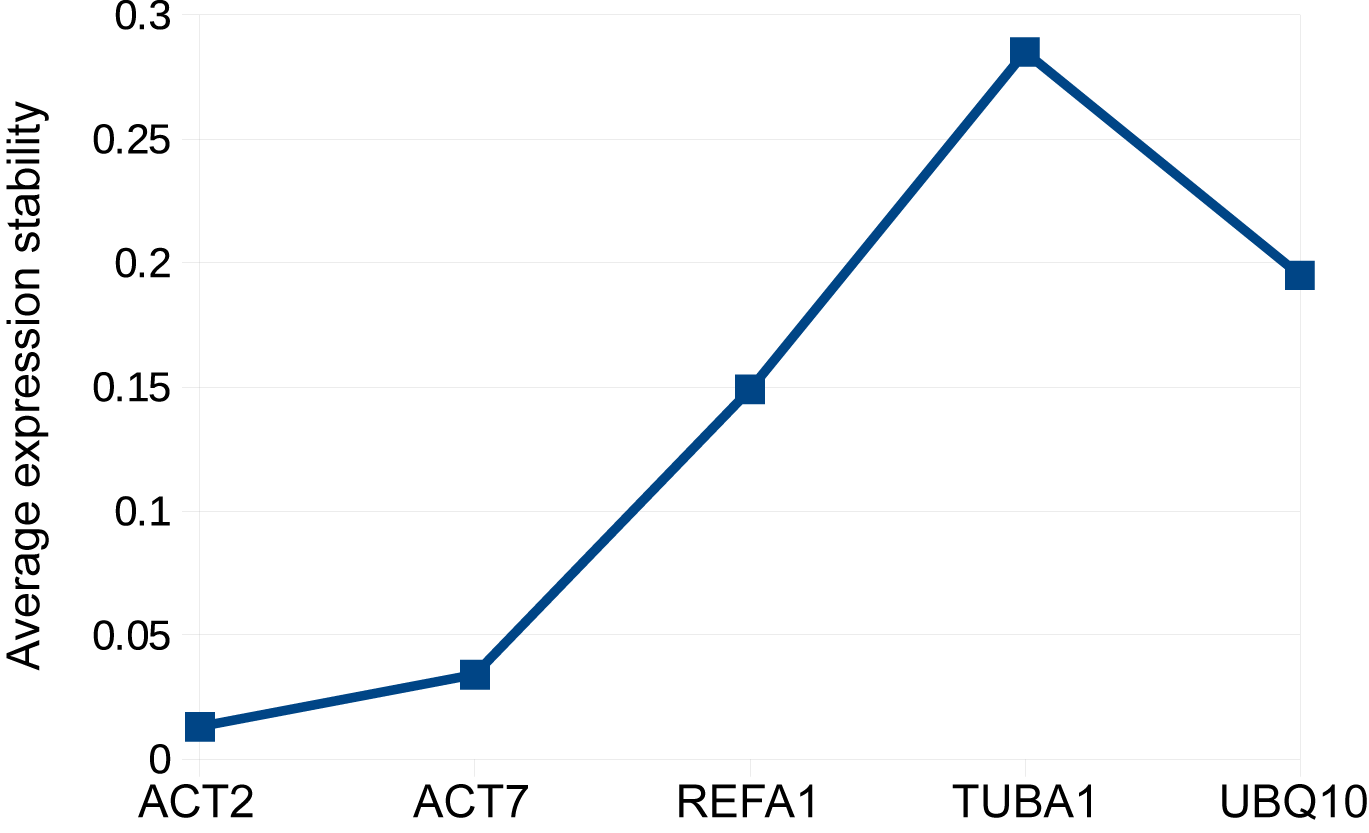

Supplement: S3 Fig — ACT2: Actin-2; ACT7: Actin-7; REFA1: Elongation factor 1-alpha; TUBA1: Tubulin alpha-1 chain; UBQ10: Polyubiquitin 10. (TIF) [file pone.0160338.s003.tif]
