# Supplementary material for: Transcriptome Analysis of Storage Roots and Fibrous Roots of the Traditional Medicinal Herb Callerya speciosa (Champ.) ScHot
Source: PLoS One. 2016 Aug 3;11(8):e0160338. doi: 10.1371/journal.pone.0160338 (PMC4972434; doi:10.1371/journal.pone.0160338)
Supplement: S2 Table — (DOC) [file pone.0160338.s005.doc]

**S2Table. Putative DEGs involve in storage root formation.**

| *Unigene* | *Log2 FC* | *Probability* | *Homologue ID* | *Evalue* | *Homologue name* |
| --- | --- | --- | --- | --- | --- |
| **Flowering related genes** | | | | | |
| Unigene18529_All | 1.467877888 | 0.836090904 | gi|402793918|ref|NM_001254508.2| | 0 | *TERMINAL FLOWER 1-like* |
| Unigene21354_All | 2.406264673 | 0.813475869 | gi|255046080|gb|FJ573245.1| | 1e-95 | *Flowering locus T* |
| CL11129.Contig2_All | -3.835191638 | 0.91581859 | gi|356509645|ref|XM_003523509.1| | 1e-90 | *probable salt tolerance-like protein At1g75540-like* |
| Unigene41170_All | 3.367286014 | 0.922707763 | sp|Q96502|COL2_ARATH | 8e-109 | *Zinc finger protein CONSTANS-LIKE 2* |
| CL2676.Contig1_All | 1.109845902 | 0.800617417 | sp|Q9FHH8|COL5_ARATH | 2e-100 | *Zinc finger protein CONSTANS-LIKE 5* |
| CL12813.Contig1_All | -1.208892351 | 0.80848246 | sp|Q9SYM2|STHY_ARATH | 1e-75 | *Probable salt tolerance-like protein At1g78600* |
| CL11129.Contig1_All | -2.496103122 | 0.887653562 | sp|Q9LQZ7|STHX_ARATH | 2e-63 | *Probable salt tolerance-like protein At1g75540* |
| Unigene41076_All | -3.914585917 | 0.92447679 | sp|O04211|ELF4_ARATH | 1e-25 | *Protein* *EARLY FLOWERING 4* |
| Unigene32835_All | -1.857608094 | 0.865447252 | sp|Q0E1D7|FLP3_ORYSJ | 2e-40 | *Flowering-promoting factor 1-like protein 3* |
| CL5099.Contig5_All | -1.682857541 | 0.852327934 | sp|Q0E1D7|FLP3_ORYSJ | 7e-36 | *Flowering-promoting factor 1-like protein 3* |
| Unigene18836_All | -10.39678671 | 0.990827461 | sp|O23624|FPF1_ARATH | 2e-40 | *Flowering-promoting factor 1* |
| **Root development related genes** | | | | | |
| CL2397.Contig6_All | 2.590338243 | 0.890583283 | gi|19880035|gb|AAM00231.1|AF358431_1 | 0 | *root-specific chalcone synthase* |
| Unigene17049_All | 2.113463815 | 0.876914774 | sp|Q9SZF7|SHR_ARATH | 9e-121 | *Protein SHORT-ROOT* |
| CL12571.Contig3_All | 2.085906729 | 0.873347148 | sp|Q9SZF7|SHR_ARATH | 4e-170 | *Protein SHORT-ROOT* |
| Unigene1621_All | 1.867199885 | 0.862723478 | sp|Q39204|RAP1_ARATH | 4e-172 | *MYC2* |
| Unigene1622_All | 1.482183612 | 0.831197827 | sp|Q39204|RAP1_ARATH | 5e-177 | *MYC2* |
| Unigene17802_All | 2.747175261 | 0.906491855 | sp|Q39204|RAP1_ARATH | 1e-118 | *MYC2* |
| Unigene41480_All | 3.119701282 | 0.917235924 | sp|Q9SHE9|LBD4_ARATH | 2e-63 | *LOB domain-containing protein 4* |
| Unigene22214_All | 2.466993703 | 0.895879803 | sp|Q8L8Q3|LBD25_ARATH | 6e-53 | *LOB domain-containing protein 25* |
| Unigene13066_All | -1.5908907 | 0.840120062 | sp|Q9AT61|LBD13_ARATH | 7e-67 | *LOB domain-containing protein 13* |
| CL3386.Contig2_All | -1.616859564 | 0.847699948 | sp|Q9M886|LBD41_ARATH | 4e-83 | *LOB domain-containing protein 41* |
| CL7515.Contig2_All | -1.860980402 | 0.865621515 | sp|Q9SN23|LBD38_ARATH | 1e-70 | *LOB domain-containing protein 38* |
| CL11371.Contig1_All | -1.946919018 | 0.86297167 | sp|Q84WP6|NAC43_ARATH | 2e-114 | *NAC domain-containing protein 43* |
| Unigene13408_All | -2.442527483 | 0.887900698 | sp|Q84TE6|NAC22_ARATH | 2e-105 | *NAC domain-containing protein 21/22* |
| CL5311.Contig4_All | -4.72798948 | 0.933633487 | sp|O49255|NAC29_ARATH | 5e-87 | *NAC transcription factor 29* |
| CL4735.Contig5_All | -1.291282841 | 0.815367408 | sp|Q9XED8|ARFI_ARATH | 5e-112 | *Auxin response factor 9* |
| Unigene39959_All | -1.276316736 | 0.817764837 | sp|Q9ZTX8|ARFF_ARATH | 0 | *Auxin response factor 6* |
| Unigene20159_All | -1.308936433 | 0.819251876 | sp|P93024|ARFE_ARATH | 0 | *Auxin response factor 5* |
| CL3373.Contig19_All | -1.367416132 | 0.817423705 | sp|Q9ZTX9|ARFD_ARATH | 0 | *Auxin response factor 4* |
| CL9882.Contig2_All | -1.188029711 | 0.806054404 | sp|Q653H7|ARFR_ORYSJ | 2e-56 | *Auxin response factor 18* |
| **Cell wall loosening related genes** | | | | | |
| CL12981.Contig1_All | -10.85474374 | 0.993212216 | sp|O23547|EXLB1_ARATH | 3e-89 | *Expansin-like B1* |
| CL14227.Contig1_All | -4.782267133 | 0.932095753 | sp|Q38865|EXPA6_ARATH | 1e-130 | *Expansin-A6* |
| Unigene25102_All | 4.7176315 | 0.938405108 | sp|O80622|EXP15_ARATH | 2e-116 | *Expansin-A15* |
| CL3091.Contig7_All | 2.983223115 | 0.909582108 | sp|O80622|EXP15_ARATH | 1e-70 | *Expansin-A15* |
| CL3091.Contig8_All | 2.833028851 | 0.904569688 | sp|Q9LDR9|EXP10_ARATH | 7e-49 | *Expansin-A10* |
| CL3091.Contig6_All | 3.080700139 | 0.916086848 | sp|Q9C554|EXPA1_ARATH | 6e-71 | *Expansin-A1* |
| Unigene41911_All | -2.869060624 | 0.893996713 | sp|Q8LDW9|XTH9_ARATH | 3e-57 | *Xyloglucan endotransglucosylase/hydrolase protein 9* |
| Unigene40780_All | -3.846239171 | 0.922291646 | sp|Q38857|XTH22_ARATH | 4e-85 | *Xyloglucan endotransglucosylase/hydrolase protein 22* |
| CL13489.Contig1_All | -4.178107889 | 0.923414318 | sp|Q9ZSU4|XTH14_ARATH | 9e-38 | *Xyloglucan endotransglucosylase/hydrolase protein 14* |
| CL1947.Contig6_All | -1.504016405 | 0.827194016 | sp|Q38909|XTH28_ARATH | 6e-134 | *Probable xyloglucan endotransglucosylase/hydrolase protein 28* |
| CL7466.Contig3_All | -2.068812122 | 0.875614671 | sp|Q9SJL9|XTH32_ARATH | 3e-123 | *Probable xyloglucan endotransglucosylase/hydrolase protein 32* |
| Unigene37972_All | -1.244757983 | 0.807753726 | sp|Q9C9Q8|PMTT_ARATH | 5e-45 | *Probable pectin methyltransferase QUA2* |
| Unigene21277_All | -1.420305425 | 0.821244804 | sp|Q9LXK7|PME32_ARATH | 2e-139 | *Probable pectinesterase/pectinesterase inhibitor 32* |
| CL11485.Contig2_All | -1.706824869 | 0.855194287 | sp|O81301|PME40_ARATH | 8e-161 | *Probable pectinesterase/pectinesterase inhibitor 40* |
| CL9924.Contig2_All | -1.779751466 | 0.854950319 | sp|Q43111|PME3_PHAVU | 0 | *Pectinesterase 3* |
| Unigene17922_All | -1.848104679 | 0.863072003 | sp|Q9FK05|PME61_ARATH | 8e-71 | *Probable pectinesterase/pectinesterase inhibitor 61* |
| Unigene36964_All | -1.91614517 | 0.856509176 | sp|O04887|PME2_CITSI | 0 | *Pectinesterase 2* |
| CL9508.Contig4_All | -3.245048775 | 0.92102217 | sp|Q1JPL7|PME18_ARATH | 7e-172 | *Pectinesterase/pectinesterase inhibitor 18* |
| CL11485.Contig3_All | -4.466230535 | 0.926086341 | sp|O81301|PME40_ARATH | 2e-106 | *Probable pectinesterase/pectinesterase inhibitor 40* |
| Unigene66068_All | -3.759721974 | 0.903984589 | sp|Q38890|GUN25_ARATH | 8e-123 | *Endoglucanase 25* |
| CL9596.Contig1_All | 3.275602211 | 0.922434224 | sp|Q9FS16|EXTN3_ARATH | 1e-32 | *Extensin-3* |
| CL9470.Contig2_All | -1.517957143 | 0.821949246 | sp|Q9T0K5|LRX3_ARATH | 0 | *Leucine-rich repeat extensin-like protein 3* |
| CL13561.Contig2_All | -2.721662097 | 0.896394141 | sp|O65375|LRX1_ARATH | 4e-32 | *Leucine-rich repeat extensin-like protein 1* |
| Unigene33730_All | -2.896312045 | 0.913110658 | sp|Q9LUI1|LRX6_ARATH | 7e-15 | *Leucine-rich repeat extensin-like protein 6* |
| Unigene16895_All | -2.911979472 | 0.898040657 | sp|Q9LJ64|PLRX1_ARATH | 1e-111 | *Pollen-specific leucine-rich repeat extensin-like protein 1* |
| CL13561.Contig1_All | -3.590811838 | 0.926325028 | sp|Q9M1G9|EXTN2_ARATH | 2e-103 | *Extensin-2* |
| Unigene1149_All | -5.755281766 | 0.94859893 | sp|Q9SN46|LRX5_ARATH | 2e-31 | *Leucine-rich repeat extensin-like protein 5* |
| Unigene26568_All | -1.662003252 | 0.845570778 | sp|O81081|LAC2_ARATH | 9.00E-23 | *Laccase-2* |
| Unigene47867_All | -9.019655588 | 0.975263189 | sp|Q9FLB5|LAC12_ARATH | 2.00E-64 | *Laccase-12* |
| CL3996.Contig1_All | -3.934814097 | 0.921552351 | sp|Q9SIY8|LAC5_ARATH | 3.00E-145 | *Laccase-5* |
| Unigene13230_All | -2.554281151 | 0.898324758 | sp|Q9SR40|LAC7_ARATH | 2.00E-104 | *Laccase-7* |
| Unigene58553_All | -2.564088775 | 0.895477416 | sp|Q9ZRF1|MTDH_FRAAN | 1.00E-96 | *Probable mannitol dehydrogenase* |
| Unigene37208_All | -2.158805835 | 0.87266594 | sp|O82515|MTDH_MEDSA | 0 | *Probable mannitol dehydrogenase* |
| **Sugar related genes** | | | | | |
| CL13177.Contig1_All | 7.676970747 | 0.969675175 | sp|Q00081|GLGL1_SOLTU | 0 | *Glucose-1-phosphate adenylyltransferase large subunit 1* |
| CL10402.Contig2_All | 6.264433124 | 0.953125528 | sp|P52417|GLGS2_VICFA | 0 | *Glucose-1-phosphate adenylyltransferase small subunit 2* |
| CL14063.Contig6_All | 4.805001865 | 0.939578475 | sp|P53536|PHSL_VICFA | 0 | *Alpha-1,4 glucan phosphorylase L isozyme, chloroplastic/amyloplastic* |
| CL12657.Contig1_All | 3.97013026 | 0.933002974 | sp|Q43092|SSG1_PEA | 0 | *Granule-bound starch synthase 1, chloroplastic/amyloplastic* |
| CL10444.Contig2_All | 2.351379228 | 0.886854067 | sp|Q0WVX5|SSY4_ARATH | 3e-103 | *Probable starch synthase 4, chloroplastic/amyloplastic* |
| CL11429.Contig2_All | -1.280885959 | 0.815525829 | sp|Q8LCP6|GUN10_ARATH | 0 | *Endoglucanase 10* |
| CL13908.Contig14_All | 4.335879064 | 0.825064847 | sp|Q9LZS3|GLGB2_ARATH | 0 | *1,4-alpha-glucan-branching enzyme 2-2, chloroplastic/amyloplastic* |
| CL886.Contig15_All | 7.343398849 | 0.928710838 | sp|P55231|GLGL3_ARATH | 4e-89 | *Glucose-1-phosphate adenylyltransferase large subunit 3, chloroplastic* |
| CL14396.Contig5_All | 4.665488306 | 0.93458929 | sp|Q94AZ2|STP13_ARATH | 0 | *Sugar transport protein 13* |
| CL1456.Contig8_All | 1.190381733 | 0.80763227 | sp|Q9SEK3|HXK1_SPIOL | 0 | *Hexokinase-1* |
| CL6463.Contig3_All | 3.836332828 | 0.928028575 | sp|Q9LPS1|HXK3_ARATH | 0 | *Hexokinase-3* |
| CL9768.Contig4_All | 3.855858415 | 0.917584449 | sp|Q38997|KIN10_ARATH | 1e-133 | *SNF1-related protein kinase catalytic subunit alpha KIN10* |
| CL9768.Contig3_All | 2.961163441 | 0.889714083 | sp|Q38997|KIN10_ARATH | 2e-156 | *SNF1-related protein kinase catalytic subunit alpha KIN10* |
| Unigene59772_All | -6.529456584 | 0.831999434 | sp|P92958|KIN11_ARATH | 5e-66 | *SNF1-related protein kinase catalytic subunit alpha KIN11* |
| Unigene59262_All | -8.080497781 | 0.940236448 | sp|P92958|KIN11_ARATH | 9e-31 | *SNF1-related protein kinase catalytic subunit alpha KIN11* |
| CL4742.Contig10_All | 2.805577109 | 0.854160964 | sp|Q9SYM4|TPS1_ARATH | 0 | *Alpha,alpha-trehalose-phosphate synthase [UDP-forming] 1* |
| CL4742.Contig7_All | 1.853849633 | 0.805581889 | sp|Q9SYM4|TPS1_ARATH | 0 | *Alpha,alpha-trehalose-phosphate synthase [UDP-forming] 1* |
| Unigene25815_All | -1.209940777 | 0.813002721 | sp|Q9LMI0|TPS7_ARATH | 9e-20 | *Probable alpha,alpha-trehalose-phosphate synthase [UDP-forming] 7* |
| CL9903.Contig2_All | 1.880860939 | 0.868669522 | sp|Q9FUD3|BZIP9_ARATH | 8e-52 | *Basic leucine zipper 9* |
| Unigene33406_All | -3.262320928 | 0.920374759 | sp|B9DGI8|BZP63_ARATH | 1e-10 | *Basic leucine zipper 63* |
| Unigene1547_All | -3.655271152 | 0.924064897 | sp|B9DGI8|BZP63_ARATH | 4e-12 | *Basic leucine zipper 63* |
| CL6174.Contig7_All | 1.380674961 | 0.831662527 | sp|Q9LXL5|SUS4_ARATH | 0 | *Sucrose synthase 4* |
| CL6174.Contig5_All | -1.830796755 | 0.86100937 | sp|O24301|SUS2_PEA | 0 | *Sucrose synthase 2* |
| CL11300.Contig1_All | 1.980317906 | 0.874638802 | sp|Q43876|SPSA_VICFA | 0 | *Probable* *sucrose-phosphate synthase* |
| CL11300.Contig2_All | 1.6682282 | 0.852001588 | sp|Q43876|SPSA_VICFA | 0 | *Probable sucrose-phosphate synthase* |
| Unigene9680_All | 2.201624383 | 0.88780459 | sp|P10290|MYBC_MAIZE | 7e-43 | *Anthocyanin regulatory C1* |
| **Phytohormone related genes** | | | | | |
| Unigene15036_All | -2.579916055 | 0.853907913 | sp|Q8L883|LAX5_MEDTR | 2e-98 | *Auxin transporter-like protein 5* |
| CL13944.Contig2_All | -1.72223663 | 0.849766805 | sp|Q9FEL6|LAX3_MEDTR | 0 | *Auxin transporter-like protein 3* |
| CL3662.Contig3_All | -1.559716397 | 0.826277291 | sp|Q9FEL8|LAX1_MEDTR | 0 | *Auxin transporter-like protein 1* |
| CL13944.Contig1_All | -2.794384057 | 0.900837304 | sp|Q9FEL7|LAX2_MEDTR | 0 | *Auxin transporter-like protein 2* |
| Unigene32833_All | -1.716068268 | 0.847765428 | sp|Q5VQY3|PIN5_ORYSJ | 4e-75 | *Probable auxin efflux carrier component 5* |
| CL14295.Contig3_All | -3.86393845 | 0.665150056 | sp|Q7G9P4|ALDO3_ARATH | 5e-178 | *Abscisic-aldehyde oxidase* |
| CL3839.Contig1_All | 2.397279496 | 0.892414622 | sp|Q9XIW0|CIPK7_ARATH | 1e-125 | *CBL-interacting serine/threonine-protein kinase 7* |
| Unigene10797_All | -3.985619154 | 0.918579328 | sp|Q8RUN2|LOG1_ARATH | 1e-84 | *Cytokinin riboside 5'-monophosphate phosphoribohydrolase LOG1* |
| CL3839.Contig2_All | 1.345807289 | 0.826089298 | sp|Q9XIW0|CIPK7_ARATH | 6e-139 | *CBL-interacting serine/threonine-protein kinase 7* |
| CL3839.Contig3_All | 2.544497555 | 0.902195495 | sp|Q9SUL7|CIPK4_ARATH | 5e-140 | *CBL-interacting serine/threonine-protein kinase 4* |
| Unigene25019_All | 1.655865439 | 0.855610404 | sp|Q94ID2|IPT5_ARATH | 9e-92 | *Adenylate isopentenyltransferase 5* |
| CL7968.Contig2_All | -1.423300827 | 0.820763206 | sp|Q9SVU0|YUC8_ARATH | 3e-170 | *Flavin-containing monooxygenase* *YUCCA8* |
| CL7102.Contig7_All | -3.058361221 | 0.905213931 | sp|Q9LSQ4|GH36_ARATH | 0 | *Indole-3-acetic acid-amido synthetase GH3.6* |
| CL5334.Contig1_All | -3.218463069 | 0.911505331 | sp|Q9SKE2|JAR1_ARATH | 8e-120 | *Jasmonic acid-amido synthetase JAR1* |
| CL5334.Contig3_All | -1.172802654 | 0.811197785 | sp|Q6I581|GH35_ORYSJ | 0 | *Probable indole-3-acetic acid-amido synthetase GH3.5* |
| CL12239.Contig1_All | -6.838853327 | 0.94602724 | sp|O82333|GH31_ARATH | 0 | *Probable indole-3-acetic acid-amido synthetase GH3.1* |
| Unigene25664_All | -3.277968237 | 0.913647175 | sp|P31531|1A1C_SOYBN | 7e-48 | *1-aminocyclopropane-1-carboxylate synthase* |
| Unigene17333_All | -3.063248019 | 0.902679205 | sp|Q0WPW4|ACCO5_ARATH | 5e-103 | *1-aminocyclopropane-1-carboxylate oxidase 5* |
| CL8934.Contig2_All | -7.326044917 | 0.951277291 | sp|Q9XG83|G2OX_PHACN | 2E-158 | *Gibberellin 2-beta-dioxygenase* |
| CL8934.Contig2_All | -7.326044917 | 0.951277291 | sp|Q9XG83|G2OX_PHACN | 2E-158 | *Gibberellin 2-beta-dioxygenase* |
| CL4188.Contig5_All | -2.216348371 | 0.884665754 | sp|Q9SQ80|G2OX1_PEA | 4E-162 | *Gibberellin 2-beta-dioxygenase 1* |
| CL1626.Contig2_All | -4.774778548 | 0.921596708 | sp|Q39111|GAOX2_ARATH | 6E-145 | *Gibberellin 20 oxidase 2* |
