# Supplementary material for: Transcriptome Analysis of Storage Roots and Fibrous Roots of the Traditional Medicinal Herb Callerya speciosa (Champ.) ScHot
Source: PLoS One. 2016 Aug 3;11(8):e0160338. doi: 10.1371/journal.pone.0160338 (PMC4972434; doi:10.1371/journal.pone.0160338)
Supplement: S3 Table — (DOC) [file pone.0160338.s006.doc]

**S3 Table. Summary of qRT-PCR validation.**

| Gene name | Forward primer 5’-3’ | Length of forward primer | Reverse primer 5’-3’ | Length of Reverse primer | Primer efficiency | Amplication length | Relatevie expression level of storage root | Sd | Relatevie expression level of fibrous root | Sd |
| --- | --- | --- | --- | --- | --- | --- | --- | --- | --- | --- |
| Unigene25102_All | TCCATTGCCACAAACGACTC | 20 | CCAGAAGCATCACTTCCACCATA | 23 | 87.39% | 148 | 5.436 | 0.4504 | 1.002 | 0.0797 |
| Unigene27068_All | AGATGAGATGGGGTGGAGTCA | 21 | ATGGGCACTTGTTCCACCAAT | 21 | 97.40% | 198 | 0.278 | 0.0309 | 1.169 | 0.6995 |
| Unigene18836_All | CTCAGATGGAAGGCAAGGGAA | 21 | TGGAATTGGTACAAGTCAGGGTC | 23 | 102.55% | 170 | 0.167 | 0.0236 | 1.013 | 0.1941 |
| Unigene41170_All | ACCACCGTGTCCCCATTCT | 19 | ACCCATTACCCTGCTGATTTT | 21 | 93.17% | 228 | 2.668 | 0.2881 | 1.006 | 0.1355 |
| Unigene25019_All | ACAACCAACAAGGTCACGGAG | 21 | ACGAATGAAGCACGGGGAG | 19 | 86.60% | 271 | 2.096 | 0.1193 | 1.002 | 0.0831 |
| Unigene41230_All | GGAGTAGTTGTTTACGATGGTGC | 23 | GATTAGTGTAGAACTTGTGGGCTGT | 25 | 92.21% | 103 | 0.383 | 0.0199 | 1.001 | 0.0653 |
| Unigene21354_All | GTTGTTGGAGGCGTAATAGGAG | 22 | ACGATTTGGGAGGGTTTGAG | 20 | 93.44% | 122 | 2.859 | 0.2395 | 1.009 | 0.1624 |
| CL2397.Contig6_All | TCGTCGGGACCAGTTTGTAA | 20 | TTCTGCCCTCTGTGCCTTG | 19 | 91.06% | 235 | 3.418 | 0.3322 | 1.009 | 0.1662 |
| CL3091.Contig8_All | TTGGATGGCAATGTCAAGGA | 20 | AGTGGAACTGAGCACCAGTGTAG | 23 | 104.34% | 173 | 2.381 | 0.1311 | 1.001 | 0.0312 |
| CL11331.Contig2_All | ATTGCTTCCCCTATCACCCA | 20 | CTGAATACCGATTACGCCTTTC | 22 | 83.07% | 278 | 4.828 | 1.4768 | 1.009 | 0.1655 |
| CL13177.Contig2_All | CTTTAGATTGAAACTGGGGTAAGC | 24 | AAGTAAGAACAGCAGATACAGCACC | 25 | 91.37% | 287 | 6.302 | 0.2849 | 1.016 | 0.2258 |
| CL13944.Contig1_All | CAGCTTCAACGAGACAGAGCA | 21 | TTCCCAAGAGTCCATAGAATACCT | 24 | 82.73% | 233 | 0.269 | 0.0344 | 1.002 | 0.0734 |
| CL14063.Contig6_All | TCGGCATCGGTTACAGATCG | 20 | TCGGCATCGGTTACAGATCG | 21 | 91.41% | 183 | 3.165 | 0.0381 | 1.001 | 0.0595 |
| Unigene1622_All | CATCACCACAGCCGACCAA | 19 | GCCGTCAAAACCGTATTCAGATA | 23 | 93.54% | 274 | 11.052 | 1.6789 | 1.012 | 0.1907 |
| Unigene17049_All | TCTATGGATGTTAAACGAGATGGC | 24 | AGGGCTTACCTCTTGAAACTTGA | 23 | 92.37% | 199 | 5.153 | 0.3345 | 1.013 | 0.2053 |

Amplication length: length of amplification product including primer regions.
